# Patients’ awareness regarding the quality of their oral hygiene: development and validation of a new measurement instrument

**DOI:** 10.1186/s12903-022-02659-4

**Published:** 2022-12-22

**Authors:** Zdenka Eidenhardt, Sebastian Busse, Jutta Margraf-Stiksrud, Renate Deinzer

**Affiliations:** 1 Department of Medicine, Institute of Medical Psychology, Justus-Liebig-University Giessen, Klinikstr. 29, 35392 Giessen, Germany; 2 Marburg, Germany

**Keywords:** Oral hygiene, Tooth brushing, Dental plaque, Dental health surveys, Health education, Health behavior, Periodontal disease

## Abstract

**Background:**

The present research aimed to develop and validate a standardised survey instrument for the assessment of patients' awareness of the quality of their oral hygiene performance.

**Methods:**

A digital questionnaire was developed that assesses both patients' naïve self-perceptions of oral cleanliness (SPOC_n_) after tooth brushing and patients' perceptions after being informed how oral cleanliness may be captured in dentistry (SPOC_d_). Three studies (N = 56 adults, N = 66 adolescents and one of their parents, N = 24 university students) assessed the instrument’s feasibility (patient reports), reliability (internal consistency), validity (correlation with other constructs; sensitivity to manipulation of actual tooth brushing), and the correlation with actual oral cleanliness after tooth brushing.

**Results:**

All study groups accepted the questionnaire well; average answering times were less than 5 min. Cronbach’s α exceeds 0.90; correlational analyses support the discriminant validity regarding oral hygiene related self-efficacy expectations and stages of change; manipulation of oral hygiene behaviour results in the expected changes of SPOC scores. Patients’ SPOC correlate only moderately with actual oral cleanliness. The comparison between SPOC_d_ scores and actual oral cleanliness indicate that they considerably overestimate their oral hygiene performance.

**Conclusions:**

The SPOC questionnaire is an easy-to-use, well-accepted, reliable and valid instrument for the assessment of patients’ awareness of the quality of their oral hygiene for research and clinical purposes. The results of the questionnaire may help to reveal unrealistic self-perceptions of patients regarding their oral hygiene. It can raise their awareness of the need to improve their skills and/or efforts in this regard.

*Trial registration* The third study was an interventional study and was registered in the appropriate national register (www.drks.de; ID: DRKS00018781; date of registration: 12/09/2019).

**Supplementary Information:**

The online version contains supplementary material available at 10.1186/s12903-022-02659-4.

## Introduction

Dental plaque is a major cause of oral diseases such as gingivitis and periodontitis [1]. The prevention of these diseases strongly relies on effective plaque control by means of self-performed oral hygiene [2, 3]. A growing part of the population seems to understand this. In recent decades, tooth brushing has become a very well-established preventive health behaviour in many regions of the world [4–11]. Today, it may even be the most common and consistently practised health behaviour of all. This is a remarkable success of all efforts aimed at improving oral health behaviour at the population level in recent decades [12].

Nevertheless, the rates of gingivitis and periodontitis remain high [13]. This is even the case in countries such as South Korea and Germany, where far more than 80% of the adult population report brushing their teeth at least twice a day [4, 5]. In doing so, they follow current recommendations [14]. However, they apparently do not gain sufficient control over gingivitis and periodontitis. From a public health perspective, this causes concern about the effectiveness of campaigns focused on self-performed oral hygiene [12]. From the perspective of those who practice oral hygiene, however, it is essential to identify possible reasons for the lack of success in their efforts. One likely explanation could be that they lack the ability to remove plaque effectively when brushing their teeth. A growing amount of evidence supports this notion: Even after performance of oral hygiene to the best of one’s abilities and without any time limit, plaque persists to a considerable degree, especially in the region of the dentogingival junction [15–19]. Interestingly, this is not the case in dental professionals. They attain nearly perfect oral cleanliness, and their rates of gingivitis and periodontitis are correspondingly low [20]. The latter finding indicates that effective plaque control and prevention of gingivitis and periodontitis are achievable goals.

Thus, the question arises as to why people do not improve the effectiveness of their tooth brushing. At least those who brush their teeth twice a day or more often appear to be motivated to invest time and effort in oral health behaviour. According to psychological models of health behaviour, such motivation depends on several factors [21–24]. Amongst them are perceived susceptibility and severity of the disease and the perceived beneficial effects of the health behaviour and perceived barriers against it. These factors are sometimes summarized under the term “decisional balance”, the weighing of the pros and cons of a behaviour. Other factors considered important are self-efficacy expectations (i.e., the expectation that one would be able to perform the behaviour as requested) and subjective norms. Since motivation does not automatically lead to behaviour, some models explicitly focus on strategies to overcome the so-called intention behaviour gap. These models emphasize the importance of concrete behavioural plans to translate motivation into action [25, 26]. All health behaviour models also make an implicit or explicit assumption that behavioural motivation can only emerge on the background of an already existing awareness that a behavioural change would be necessary to improve or maintain one’s health.

**Fig. 1:**
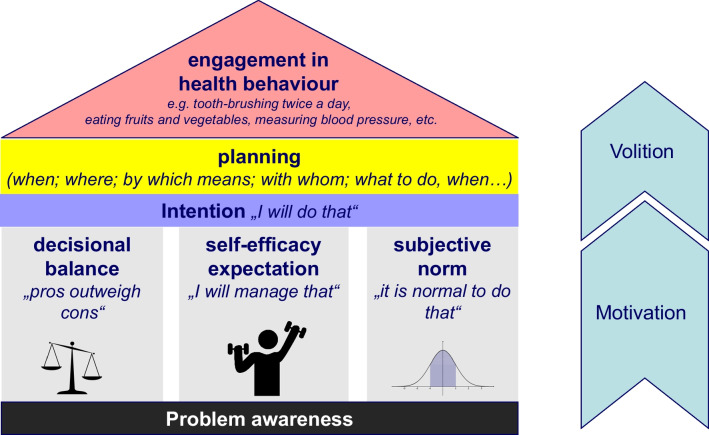
Key assumptions of health behaviour models referred to in the text. Engagement in a health behaviour prerequisites *problem awareness*, i.e. the awareness that one should do so. However, this alone does not automatically lead to behaviour or even sufficient behavioural motivation. This behavioural *motivation* is considered to be influenced by at least three aspects: (1) Weighing of pros and cons of the behaviour, i.e. *decisional balance*. (2) The expectation that one will be able to perform the behaviour as requested, i.e. *self-efficacy expectation*. (3) One’s beliefs whether it would be normal to do that and one’s willingness to fulfil norms, i.e. *subjective norms*. If the resulting motivation is strong enough, the *intention* to engage in the behaviour might emerge. This, however, will still not result automatically in behaviour. Uncertainty about how best to implement the behaviour and obstacles that get in the way can hinder *volition*. Thorough *planning* of the health behaviour, as concrete as possible, is seen as an important factor in helping to bridge the gap between intention and behaviour and to turn intention into action

**Fig. 2:**
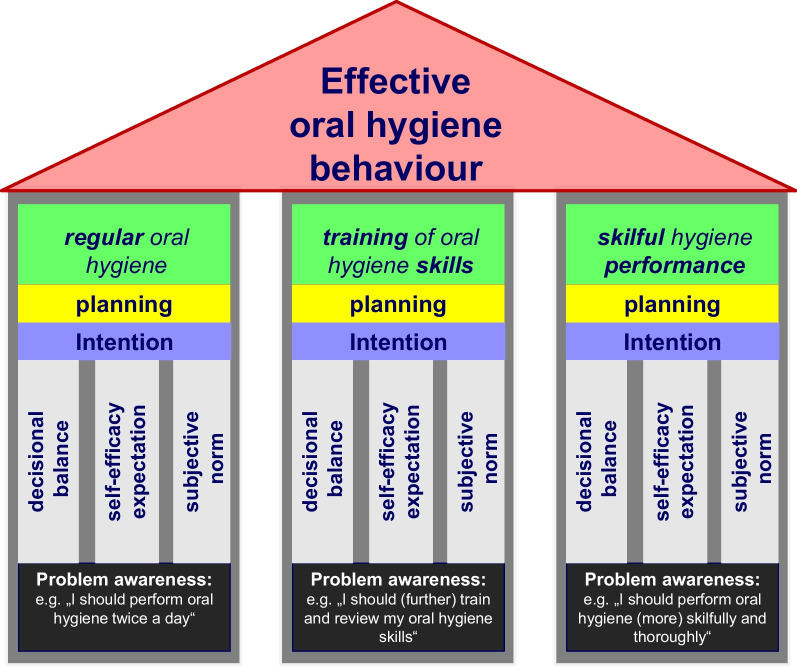
Deconstruction of effective oral hygiene behaviour into three behavioural domains (green rectangles). The general psychological constructs underlying these behaviours are the same for all domains (see also Fig. 1). However, their content differs according to the specific behaviour to which they refer. This is illustrated for problem awareness (black rectangles) but also applies to the other constructs. For a general model see also https://commons.wikimedia.org/wiki/File:Effective_health_behaviour.jpg

Figure 1 summarizes the key assumptions of the above mentioned health behaviour models. However, effective oral health behaviour and even effective tooth brushing per se are more complex than such a model suggests. One might instead deconstruct it into three distinct behavioural categories which contribute to effective oral hygiene: (1) Engagement in oral hygiene/tooth brushing, (2) engagement in acquiring the skills to remove plaque effectively, (3) engagement in skillful performance. To better understand the effectiveness of oral hygiene behaviour, one should thus consider all three behavioural categories and the respective motivational mechanism underlying them. These refer to the engagement in the behaviour as such, to the training of skills, and to the extent to which one applies the skills. Figure 2 summarizes these considerations.

The above mentioned research concerning tooth brushing abilities demonstrates that even people who engage in regular oral hygiene behaviour (cf. left side of Fig. 2) do not reach the expected endpoint (i.e., oral cleanliness). It also indicates that many people lack the skills to achieve oral cleanliness (cf. middle of Fig. 2). The question thus arises why they do not engage in improving their oral hygiene skills and performance. One reason could be that they lack an adequate awareness of their deficit in this regard. Such awareness of their deficient skills, however, would form the basis for any change in behaviour (see bottom of Fig. 2). It would therefore be important to have a measurement instrument to capture this awareness.

The present research thus aimed to develop and validate a standardized instrument to assess patients’ awareness regarding the quality of their oral hygiene performance in terms of achieved oral cleanliness. To date, no standardized instrument exists allowing such an assessment. Therefore, a questionnaire was developed which assesses patients’ self-perceived overall oral cleanliness after brushing and their estimation of the cleanliness of different areas of the dentition. Three studies assessed the feasibility, reliability and validity of the final instrument.

## General methods

To assess patients’ awareness regarding the quality of their oral hygiene performance, a standardized questionnaire assessing their self-perceived oral cleanliness (SPOC) after oral hygiene was developed and validated.

### Development of the questionnaire to assess self-perceived oral cleanliness after oral hygiene

Analogous to the Delphi method [27] the members of the working group developed the items to measure self-perceived oral cleanliness after brushing (SPOC) according to content aspects and face validity (cf. [28] on the methods of questionnaire design). Afterwards, dental laypersons judged the understandability and feasibility of the first drafts of the questionnaire. Their feedback led to the current state of the items in terms of their presentation and type of wording of the items and the answer format. Additional file 1 shows the screenshots of the questionnaire pages and their translation into English.

**Fig. 3:**
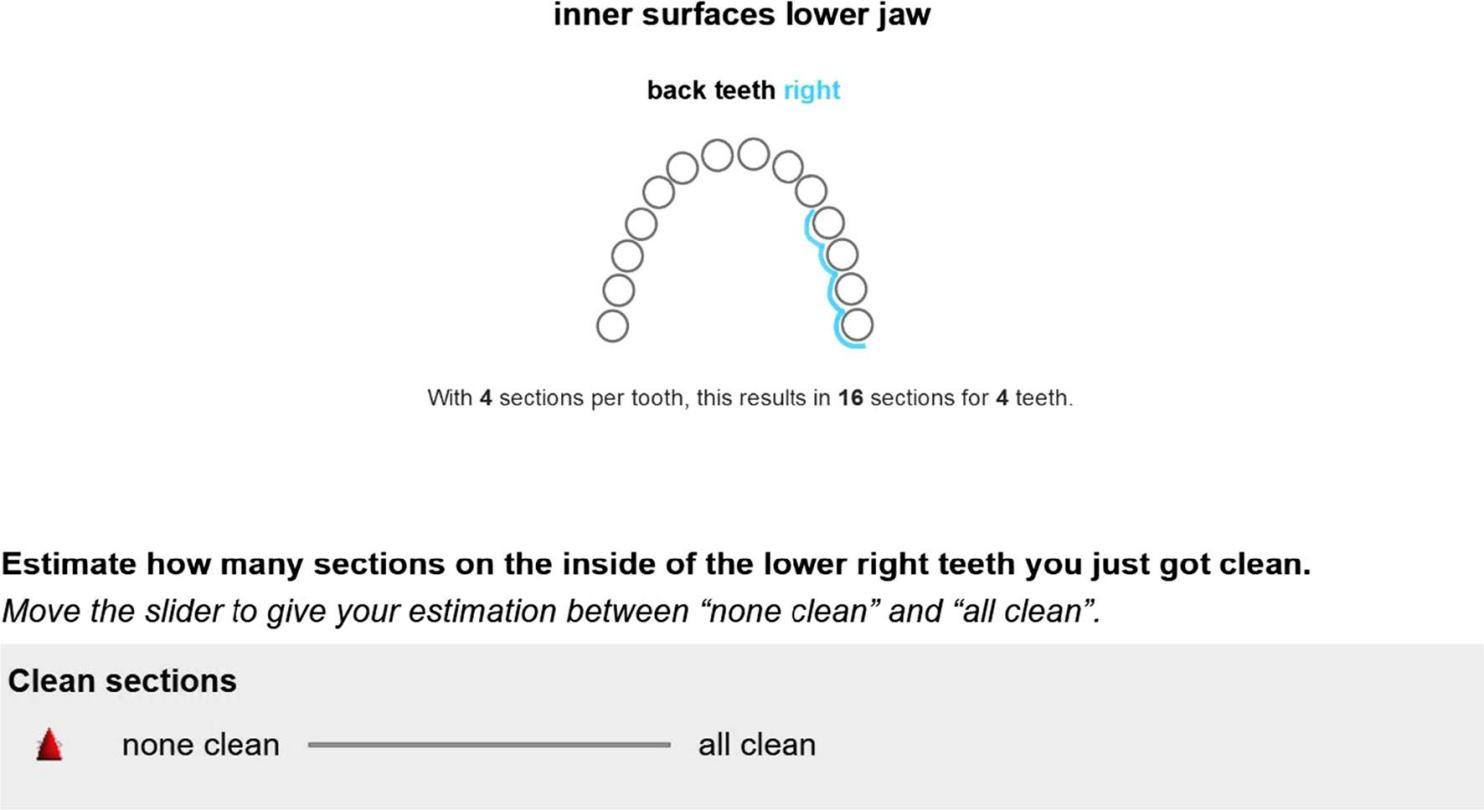
Example of one of the 12 items of the SPOC_d_ scale translated into English. The 12 items refer to the inner and outer surfaces of the 6 sextants. Participants place the red triangle between "none clean" and "all clean" to indicate their estimation. Before answering the items, participants read an explanation of the MPI (for details, see text and Additional file 1)

The first item of the questionnaire assesses self-perceived overall oral cleanliness after performing oral hygiene. This item should represent patients’ naïve perception of their oral cleanliness (SPOC_n_). A second set of items aims at the patients’ self-perceived oral cleanliness based on the standards applied by a dentist. Previous research of our group indicates that patients’ subjective understanding of oral cleanliness might differ from that of their dentists. This requires an explanation of how a dentist would assess oral cleanliness. Thus, patients learn through an illustrated written explanation how the dentist applies a simple plaque index (MPI; [29]). This index assesses the presence or absence of plaque at the dento-gingival junction. The gum line of a tooth is divided into 8 sections (4 per surface). Each section receives either a score of 0 (clean) or 1 (plaque). After the explanation, patients estimate the achieved oral cleanliness in each of the 12 areas of their dentition (inner and outer surfaces of the six sextants). Figure 3 shows an example item. The mean of the 12 items represents the patients’ self-perceived overall oral cleanliness based on the standards of a dentist (SPOC_d_). The assessment of the 12 distinct areas also allows more specific evaluations, e.g., patients’ SPOC_d_ of their inner vs. outer surfaces.

The resulting questionnaire is implemented via a (tablet) computer using the survey software SoSci Survey [30], which also provides information about the time participants spend answering the questionnaire. It also permits the establishment of a fixed sequence in which patients read the questions and their respective explanations and respond to them. The sequence in which they rate the 12 areas varies according to a programmed random shuffle algorithm in SoSci Survey. The answering format of all items is a visual analogue scale. Thus, patients adjust a slider, which represents a value of 0–100, whereby a higher value also means a higher self-perceived oral cleanliness.

### Validation studies

After the development of the questionnaire, three separate studies assessed its validity. Study 1 examined feasibility, internal consistency (as an indicator of reliability [31]) and discriminant (divergent) validity with respect to oral hygiene related self-efficacy expectations [32], which represent a different though acquainted construct (see Fig. 1). Study 2 assessed the same parameters in two other populations. It also proved the discriminant validity with regard to the stage of change [24] of daily oral hygiene. Furthermore, it examined the relationship to actual plaque levels and the tooth brushing behaviour of the participants. This study also added three items assessing the participants’ comprehension of the SPOC_d_ items and analysed how this might have affected the results. Study 3 assessed the sensitivity of the questionnaire regarding differences in actual tooth brushing behaviour by means of a randomized controlled trial (RCT).

### Data analysis

All analyses were computed by IBM SPSS Statistics 28 (IBM Corporation, U.S.A). Normal distribution assumption was tested using the Kolmogorov–Smirnov test and visual inspection. The assumption was rejected if the test showed a significant result (*p* ≤ 0.05) and visual inspection revealed a significant deviation from the normal distribution [33].

## Study 1: feasibility, internal consistency and discriminant validity of the questionnaire

### Method

#### Ethics

The study protocol was conducted according to the principles of the Declaration of Helsinki. Participants received an e-mail inviting them to fill in a survey after they had brushed their teeth. They were informed that their participation would be voluntary and anonymous, that the sender of the e-mail would not know whether they participated or not, and that their participation was not related to any advantages or disadvantages.

#### Main procedure

N = 56 (14 male, 42 female; aged between 19 to 65 years, M = 30.5 ± 12.0) friends and family (without education in dentistry) of the employees of the Institute of Medical Psychology in Gießen volunteered to answer the survey after brushing their teeth. The survey consisted of a German translation and adaptation of the scale of Stewart et al. [32] to assess oral hygiene related self-efficacy expectations (OHSEE; items shown in Additional file 2) and of the questionnaire assessing SPOC. Finally, the participants indicated their age and gender. Together with the invitation to participate, they were also encouraged to provide critical feedback on the questionnaire regarding its comprehensibility and the effort involved in answering it.

#### Statistical analyses

This design allows for the assessment of three important parameters characterizing the SPOC questionnaire. A close to zero correlation between the OHSEE and the two scales of the SPOC questionnaire (SPOC_n_ and SPOC_d_) would indicate a good discriminant validity of the SPOC questionnaire with respect to oral hygiene related self-efficacy expectations. Thus, these correlations are computed to test the discriminant validity. The internal consistency of the SPOC_d_ informs about the reliability of this scale and is therefore computed as a measure for the reliability of the scale. The correlation between the two SPOC scales and a t-test for dependent measures to compare their means is computed to estimate the degree of convergence between the naïve standards of the patients regarding oral cleanliness to those reconsidered according the approach of the dentist. Statistical analyses thus comprise correlations (Pearson correlations if the normal distribution assumption is acceptable, Spearman correlations if it is violated) and the assessment of the internal consistency via Cronbach’s α along with the item selectivity of the SPOC items together with univariate statistics (means, standard deviations). The calculation with G*Power [34] showed that a sample size of N = 40 would be sufficient to detect correlations of r ≥ 0.4 and effect sizes of differences between dependent measures of d ≥ 0.4 with α = 5% and 1 − β = 0.80.

### Results

#### Feasibility

All participants reported that the questionnaire was comprehensible and that there were no problems in answering the questions.

#### SPOC descriptives, item analysis and internal consistency

The descriptive scores of the naïve self-perception of oral cleanliness (SPOC_n_) and various aggregated SPOC_d_ scores are shown in Table 1. Means and standard deviations of the 12 items assessing the SPOC_d_ score ranged from M = 70.1 to M = 87.4 and SD = 11.0 to SD = 23.0. Consistently, negative skewness signs indicated a left-skewed distribution. With one exception, the kurtosis values were consistently positive, indicating more pronounced marginal areas of the distribution compared to the normal distribution. The item-total correlations ranged from r = 0.51 to r = 0.87. Analysis of these data revealed excellent internal consistency (Cronbach’s alpha, α = 0.938). Detailed information about the values on the individual SPOC_d_ items is shown in Additional file 2.

**Table 1:** Descriptives of SPOC_n_ and various aggregated SPOC_d_ scores (N = 56)

Item	Min	Max	*M*	*SD*	Skew	Kurtosis
SPOC_n_	38	100	77.5	12.3	− 1.118	1.792
SPOC_d_						
Total	38.2	99.1	76.7	13.2	− 0.724	0.708
Outer	42.2	98.2	82.0	11.1	− 0.943	1.645
Inner	26.8	100	71.5	16.7	− 0.639	0.284
Maxilla	37.2	100	76.2	13.2	− 0.513	0.247
Mandible	38.3	99.5	77.3	14.0	− 0.861	0.833

SPOC: Self-perceived oral cleanliness. SPOC_n_: Naïve overall SPOC before being informed within the questionnaire about the standards of a dentist. SPOC_d_: SPOC after being informed about the standards of a dentist. Total: SPOC_d_: Mean of the SPOC_d_-scores for all 12 areas of the dentition (sextants by inner vs. outer surfaces). Outer/inner: SPOC_d_: Mean of the SPOC_d_-scores for outer/inner surfaces. Maxilla/mandible: Mean of the SPOC_d_-Scores for the inner and outer surfaces of the maxilla/mandible

#### Relationship between naïve SPOC (SPOC_n_) and SPOC based on the standards of a dentist (SPOC_d_)

The correlation between SPOC_n_ and SPOC_d_ was r = 0.445 (*p* < 0.001). The paired t-test indicated no significant mean difference between SPOC_n_ and SPOC_d_ (*p* = 0.698, *d* = − 0.052).

#### Discriminant validity regarding oral hygiene related self-efficacy expectations

Tests for normality led to a rejection of the assumption of normal distribution for the OHSEE scale only. The correlations of the two SPOC scales with OHSEE scores were rho = 0.081 (SPOC_n_: *p* = 0.555) and rho = 0.256 (SPOC_d_: *p* = 0.057), respectively.

### Discussion

Partcipants’ feedback indicates the good feasibility of the SPOC questionnaire. Both the item-total correlations and the internal consistency analysis indicate an excellent reliability of the newly developed instrument. However, the good internal consistency is not surprising and should be seen as a consequence of the structure of the questionnaire. The items simply represent oral hygiene in different areas of the mouth. One would therefore expect them to be relatively well correlated with each other. Correlational analysis reveal a very good discriminant validity with respect to oral hygiene related self-efficacy expectations (OHSEE). Furthermore, the significant but only moderate correlation between SPOC_n_ and SPOC_d_ supports the consideration that the naïve perception of one’s oral cleanliness is not fully consistent with the self-perceived oral cleanliness based on the standards applied by a dentist. In summary, the results of Study 1 support the assumption that the SPOC questionnaire is reliable and has good discriminant validity with respect to the OHSEE. The study also shows that the two scales of the SPOC assess differing although interrelated constructs. However, skewness and kurtosis revealed that the participants tended to rate their oral cleanliness after brushing rather high. To ensure that this would not reflect a misinterpretation of the questions, the questionnaire was supplemented with three comprehension questions (see Additional file 1: pp. 24–27) to check whether the participants had understood the explanation of the cleanliness measurement and its query. A second study examined the resulting expanded questionnaire and explored it’s reliability and validity in two other samples.

## Study 2: comprehensiveness of the questionnaire and its relationship to parameters of tooth brushing behaviour and actual oral cleanliness after brushing in adults and adolescents

The first study brought about promising results in an unselected sample of adults. The second study tested the instrument in two other groups, i.e., adolescents and their parents. The previous study also raised some concerns about whether all participants indeed had understood the standards of a dentist, which underlie the SPOC_d_ items. Thus, a comprehension check was added at the end of the questionnaire. To further explore the validity of the questionnaire, the discriminant validity was assessed not only regarding oral hygiene related self-efficacy expectations (OHSEE, [32]) but also regarding the stage of change (SofC, [24]) with respect to thorough daily oral hygiene. Additionally, this study explored the relationship between SPOC and the actual oral cleanliness assessed by a dentist as well as the relationship to the actual tooth brushing behaviour in terms of total brushing time and completeness of brushing as an indicator for criterion validity.

The major aim of the second study was thus to learn more about the comprehensibility and validity of the questionnaire in adolescents and adults.

### Method

#### Ethics

The second study was part of a larger project analysing the oral hygiene behaviour of adolescents and their parents (see [35, 36]). The study protocol was conducted according to the principles of the Declaration of Helsinki and was approved by the Ethics Board of the Medical Faculty at the University of Giessen, Germany (approval no. 255/18). All participants (children and their parents) provided informed written consent. The data of the parents and their children were collected pseudonymously, i.e., a code was assigned to each participant, and the collected data were only assigned to the code and not to the personal data of the participants.

#### Participants

For details on participant characteristics, recruitment and inclusion/exclusion criteria, see [35] and [36]. Briefly, 66 adolescents (10-year-olds [n = 42; 21 male, 21 female; M = 10.1 ± 0.5] and 15-year-olds [n = 24; 17 male, 6 female, 1 non-binary; M = 15.2 ± 0.3]) and 66 parents (11 male, 54 female, 1 non-binary, aged between 32 and 57 years, M = 44.5 ± 5.3) were recruited through schools, social media, the university's internal mailing list and print media. Every family was compensated with 50 € for their participation. Only participants with very good knowledge of the German language were included, as the questionnaires were in German.

#### Extension of the questionnaire by a comprehension check

To check participants’ comprehension of the SPOC_d_ items, three items at the end of the questionnaire were added (see Additional file 1). They depict three different situations of a tooth surface, which should result in different numbers of clean sections. The participants gave their estimation, which would be the correct number in the respective situation. Comprehension is considered if all three estimations are correct. Non-comprehension is considered if at least one estimation is wrong.

#### Main procedure and data collection

Deinzer et al. [35] and Eidenhardt et al. [36] provide a detailed description of the procedure. Briefly, the examination for parent and child took place simultaneously in separate rooms. A five-step study procedure was followed: (1) first questionnaire survey, (2) first clinical assessment, (3) instruction to brush the teeth “as thoroughly as possible so that they are completely clean” and video observation of oral hygiene behaviour, (4) second clinical assessment and (5) second questionnaire survey. The present study refers to the questionnaire data (steps 1 and 5) and the observational and clinical data from the second clinical assessment (steps 3 and 4). In the following, only the instruments relevant to this study will be described in detail here.

Oral hygiene related self-efficacy expectations (OHSEE, [32]; items shown in Additional file 2) and the stage in the course of change (SofC, [24]; items shown in Additional file 2) with regard to the statement "I keep my teeth clean every day" were assessed during the first questionnaire survey (step 1). After video observation of tooth brushing, participants' plaque was recorded using the Marginal Plaque Index (MPI, [29]), which also forms the basis of the explanation participants receive before answering the SPOC_d_ items. Participants then answered the SPOC questionnaire (step 5). During the questionnaire surveys, the participants were left alone. Directly after completing the questionnaires, the examiners asked them about any difficulties they may have had with the questionnaires.

#### Observed tooth brushing behaviour

The videos were analysed with the observation software Mangold INTERACT 18 (Mangold International GmbH, Arnsdorf, Germany). A detailed description of the observed parameters and the calibration of the observers is given by Deinzer et al. [35] and Eidenhardt et al. [36]. Of the behavioural parameters described there, this study merely considers the tooth contact time (net brushing time without spitting, rinsing, etc.) and the quality index of tooth brushing regarding brushing time in sextants (QIT-S, [15]). The QIT-S represents how extensively and comprehensively teeth are brushed at inner and outer surfaces, respectively. It can take scores from 0 to 9, while 0 means that no sextant was brushed on the respective site (inner vs. outer surfaces) by more than 1 s and 9 means that all sextants were brushed by at least 7.5 s on that site.

#### Calibration of the examiners

Prior to the assessment of the clinical parameters, the dental examiners were calibrated as described in detail in Deinzer et al. [35]. Briefly, the process of calibration was as follows: First, the dentists were instructed by an experienced examiner and then rated five participants who were not involved in the current analysis. Calibration was considered successful if more than 90% of the scorings were identical and the remaining 10% never differed by more than one score in five consecutive participants. The investigators were blinded to the participants' oral hygiene behaviour and questionnaire data. Prior to behavioural analyses, an experienced examiner trained and calibrated the observers with videos from previous studies. The calibration was considered successful if the intraclass correlations (ICCs), depending on the observation parameter, were r ≥ 0.9 after 5 or 10 observed videos, respectively. For more details regarding the calibration procedure, see [35] and [36].

#### Statistical analyses

Statistical analyses correspond to those of study 1 but were extended and refined in some respect. Additional correlational analyses in the present study refer to the stage in the course of change (SofC) as well as the actual oral cleanliness and the behavioural data. The calculation of the power for the statistical analyses with G*Power [34] showed that the realized sample size yielded a power of 1 − ß = 0.80 for the detection of small to medium effect sizes in correlational analyses and within-group comparisons. Participants whose comprehension check was negative were excluded from all analyses regarding the reliability and validity criteria of the SPOC_d_ data. To further investigate the specifics of these participants, effect sizes of comparisons to those whose check was positive were calculated. According to Cohen [37], effect sizes of d ≥|.2| |.5| |.8| are considered small, medium and large, respectively.

### Results

#### Feasibility and results of the comprehension check

Both the adolescents and their parents reported that there were no problems in answering the questions. The average time to complete the questionnaire equalled 323 ± 117 s in 10-year-olds and 253 ± 118 s in 15-year-olds and 269 ± 69 s in parents. Overall, 81% of the 10-year-olds (n = 34), 88% of the 15-year-olds (n = 21) and 82% of the parents (n = 54) had answered all three comprehension items correctly. These participants did not differ in their SPOC_d_ scores by more than small effect sizes from those who made at least one error (10-year-olds: d = − 0.164; 15-year-olds: d = 0.374; parents: d = 0.232). All further analyses were confined to those participants whose comprehension check revealed a positive result (all three items correctly answered). Additional file 2 shows the respective analyses for the whole group.

#### SPOC-descriptives, item analyses and internal consistency

Table 2 shows the descriptives of SPOC_n_ and various aggregated SPOC_d_ scores for the three age groups. As the 10- and 15 year olds with correct answers did not differ in SPOC_n_- and SPOC_d_-scores (all *p*’s ≥ 0.125), all following analyses combined them into one group. The item-total correlations of the SPOC_d_ items varied between r = 0.62 and r = 0.88 in adolescents and r = 0.70 and r = 0.87 in adults. Cronbach’s α for SPOC_d_ equaled α = 0.958 in adolescents and α = 0.954 in parents, respectively.

**Table 2:** Descriptives of SPOC_n_ and various aggregated SPOC_d_ scores of parents and adolescents

Item	10-year-olds (n = 34) *M* ± *SD*	15-year-olds (n = 21) *M* ± *SD*	Parents (n = 54) *M* ± *SD*
SPOC_n_	78.5 ± 16.4	79.8 ± 13.5	76.0 ± 18.3
SPOC_d_			
Total	71.2 ± 19.7	68.0 ± 14.5	70.1 ± 17.3
Outer	77.0 ± 18.4	74.6 ± 14.4	75.8 ± 16.9
Inner	65.5 ± 23.6	61.3 ± 18.1	64.4 ± 19.3
Maxilla	72.3 ± 19.3	69.5 ± 13.5	70.8 ± 17.9
Mandible	70.1 ± 20.6	66.4 ± 16.3	69.4 ± 17.6

SPOC: Self-perceived oral cleanliness. SPOC_n_: Naïve overall SPOC before being informed within the questionnaire about the standards of a dentist. SPOC_d_: SPOC after being informed about the standards of a dentist. Total: SPOC_d_: Mean of the SPOC_d_-scores for all 12 areas of the dentition (sextants by inner vs. outer surfaces). Outer/inner: SPOC_d_: Mean of the SPOC_d_-scores for outer/inner surfaces. Maxilla/mandible: Mean of the SPOC_d_-scores for the inner and outer surfaces of the maxilla/mandible

#### Relationship between naïve SPOC (SPOC_n_) and SPOC based on the standards of a dentist (SPOC_d_)

The correlation between SPOC_n_ and SPOC_d_ was r = 0.726 (*p* < 0.001) in adolescents and rho = 0.583 (*p* < 0.001) in parents. The paired t-test indicated a significant mean difference between SPOC_n_ and SPOC_d_ in adolescents (*p* < 0.001, d = − 0.723) and in parents (*p* = 0.019, d = − 0.328), with no such difference observed in the 11 adolescents (*p* = 0.109, d = − 0.531) and 12 parents (*p* = 0.869, d = 0.049) with a negative comprehension check.

#### Discriminant validity regarding oral hygiene related self-efficacy expectations and the stage of change of daily oral hygiene (SofC)

In adolescents, the correlations of the two SPOC scales with OHSEE scores were r < 0.35 (SPOC_n_: r = 0.337, *p* = 0.012; SPOC_d_: r = 0.218, *p* = 0.110). Tests for normality led to a rejection of the assumption of normal distribution for the SofC. The correlations of the two SPOC scales and SofC were rho < 0.19 (SPOC_n_: rho = 0.087, *p* = 0.530; SPOC_d_: rho = 0.179, *p* = 0.191). There was no difference between the adolescents who indicated that they kept their teeth clean every day for more than 6 months (n = 40) and those who did not (SPOC_n_: d = 0.276, *p* = 0.366; SPOC_d_: d = 0.374, *p* = 0.223).

In parents, the tests for normality led to a rejection of the assumption of normal distribution for all psychometric scales. The correlations of the two SPOC scales with OHSEE scores were rho < 0.24 (SPOC_n_: rho = 0.231, *p* = 0.092; SPOC_d_: rho = 0.096, *p* = 0.491). The correlations of the two SPOC scales and SofC were rho < 0.18 (SPOC_n_: rho = 0.170, *p* = 0.220; SPOC_d_: rho = 0.021, *p* = 0.881). There was no difference between the parents who indicated that they kept their teeth clean every day for more than 6 months (n = 47) and those who did not (SPOC_n_: d = 0.610, *p* = 0.138; SPOC_d_: d = − 0.037, *p* = 0.928).

#### Relationship between SPOC and actual oral cleanliness

The overall MPI values equalled M = 77.8 ± 16.7 in adolescents and M = 69.7 ± 15.3 in parents. Thus, their de facto oral cleanliness (100—MPI) was M = 22.2 ± 16.7 and M = 30.3 ± 15.3. This differed highly significant from the respective SPOC_d_ both in adolescents (paired t-test: *p* < 0.001, d = 1.877) and parents (*p* < 0.001, d = 2.211). The correlations between the de facto oral cleanliness and the two SPOC scores were rho < 0.10 for adolescents (SPOC_n_: rho = 0.098, *p* = 0.476; SPOC_d_: rho = − 0.006, *p* = 0.968) and rho > 0.37 for parents (SPOC_n_: rho = 0.500, *p* < 0.001; SPOC_d_: rho = 0.375, *p* < 0.001).

#### Relationship between SPOC and tooth brushing behaviour

In adolescents, there were no significant correlations between the two SPOC scores (SPOC_n_ and SPOC_d_) and total time of tooth brushing (rho ≤ 0.191; *p* ≥ 0.164). Also, no significant correlation emerged between the QIT-S scores for inner and outer surfaces and the respective SPOC_d_ scores (rho ≤ 0.168; *p* ≥ 0.221) or the general SPOC_d_ (rho ≤ 0.228; *p* ≥ 0.095) or SPOC_n_ score (rho ≤ 0.058; *p* ≥ 0.674).

In parents, a significant correlation was found between the SPOC_d_ score for inner surfaces and the QIT-S at inner surfaces (rho = 0.549; *p* < 0.001). This QIT-S score also correlated with SPOC_n_ (rho = 0.616; *p* < 0.001) and the general SPOC_d_ (rho = 0.520; *p* < 0.001). No significant correlations were seen between the QIT-S at outer surfaces and the respective SPOC score (rho = − 0.182, *p* = 0.189) or the two overall SPOC scores (SPOC_n_: rho = − 0.058, *p* = 0.678; SPOC_d_: rho = − 0.035; *p* = 0.804). Total tooth brushing time did not significantly correlate with SPOC_n_ (rho = 0.189, *p* = 0.172) or SPOC_d_ (rho = 0.129; *p* = 0.351).

### Discussion

The results of the current study confirm and extend the results of Study 1. They show that the internal consistency of the questionnaire is excellent not only for use with adults but also with adolescents. Furthermore, both groups indicated a good subjective comprehensibility of the questionnaire and answered in a reasonable amount of time. This indicates the good feasibility of the questionnaire in adolescents and adults. However, even though all participants indicated that they had no problems answering the questionnaire, almost 20% of the parents and adolescents did not answer all comprehension questions correctly. This indicates that the comprehension check is mandatory to come to meaningful interpretations of the results. The analyses confined to those with a positive comprehension check support the results of Study 1 that SPOC_n_ and SPOC_d_ are different although interrelated constructs. The current study extends this finding as it shows that within those who pass the comprehension test, the two assessments lead to different overall results with a higher naïve estimation of the SPOC (SPOC_n_) compared to the one informed about the standards of a dentist (SPOC_d_). This indicates that their naïve concept of oral cleanliness is less strict. The present analysis further extends the results of Study 1, as it now proves good discriminant validity not only with regard to oral hygiene related self-efficacy expectations (OHSEE) but also with regard to the stage of change (SofC). The study also indicates that the SPOC indeed differs considerably from the de facto oral cleanliness as assessed by a dentist. This supports the notion mentioned in the introduction that there might be insufficient awareness of the need to improve one’s oral hygiene skills. Interestingly, even though both adolescents and parents overestimated their oral cleanliness considerably, only parents’ estimation showed a significant correlation with the dentist’s assessment. The results also indicate that their estimation might be influenced by the extensiveness and comprehensiveness they brush their inner surfaces, while adolescents’ SPOC was not related to any of the behavioural parameters examined. However, one should keep in mind that the QIT-S at outer surfaces is close to perfect in a majority of participants and that they responded to the instruction to brush their teeth to the best of their abilities with an extended overall tooth brushing time. This might have masked relationships between SPOC and tooth brushing behaviour.

Based on the results to this point it cannot be ruled out that the SPOC estimation reflects a general assumption about the maximum achievable cleanliness rather than an estimation of the actual performance. Similarly, in Study 1, participants might have estimated the usual degree of oral cleanliness one might achieve. Thus, up to this point, it has not been clearly established whether oral hygiene behaviour actually influences SPOC scores, i.e., whether the SPOC questionnaire is sensitive to changes in oral hygiene behaviour. A third study therefore aimed to assess the validity of the instrument in this respect.

## Study 3: sensitivity of the questionnaire

Study 3 assessed by means of a randomized controlled trial (RCT) the sensitivity of the instrument to manipulation of the oral hygiene behaviour. Participants in this experiment either brushed their teeth in the lab without any disturbance or had to stop it after 1 min of brushing. Immediately afterwards, they completed the SPOC questionnaire. It was hypothesised that premature termination of tooth brushing would result in lower SPOC scores.

### Method

#### Ethics

The study protocol was conducted according to the principles of the Declaration of Helsinki and was approved by the Ethics Board of the Medical Faculty of the University of Giessen (no. 124/19). This study is registered at the German Clinical Trials Register (www.drks.de; DRKS-ID: DRKS00018781; date of registration: 12/09/2019). All participants provided informed written consent. All data collected were anonymised. Since all data were recorded with the same device (tablet computer) in the same program (SoSci Survey), pseudonymisation or noting the identity of the participants were not necessary, since the demographic data, the questionnaire data and the test conditions can be directly (anonymously) assigned to each other.

#### Participant recruitment and power calculation

The participants were recruited via the mailing list of the Justus-Liebig-University Giessen and by personal approach in the vicinity of the University. The mailing informed them about the topic of the study (tooth brushing) and the gratification they would receive (a tooth brushing kit and the participation in a raffle of 20 €; 1 of 4 tickets wins). Due to the possible distorting effects on the study results, the following exclusion criteria were applied: (1) poor knowledge of German, (2) study or profession in the dental field, and (3) prior participation in studies of the Institute for Medical Psychology. After contact had been made, the exclusion criteria were clarified, and an appointment was arranged after brief information about the procedure had been provided. The assessments took place in rooms of the Institute for Medical Psychology, Justus-Liebig-University Giessen, from December 2019 to March 2020. The initially planned sample size of 42 participants allowed for the detection of large effect sizes with a power of 1 − β = 0.80. The power calculation was performed using G*Power [34]. Due to a general lockdown in Germany because of the COVID-19 pandemic, participant recruitment stopped in March 2020 when the sample comprised 24 participants. As the ongoing pandemic and the related restrictions in human research remained unforeseeable, the study was terminated, and analysis was accomplished with a sample size of 24 participants. Sensitivity analyses revealed that the given distribution of participants to the two study groups (10:14) requires an effect size of d = 1.06 to become significant with the given power of 1 − β = 0.80 and the given α-level of α = 0.05. This was considered to be within the range of possible results and thus acceptable. A flow diagram of the recruitment process can be found in Additional file 2.

#### Randomization

For randomization, one box for female participants and one box for male participants were set up, each containing six opaque sealed envelopes with a slip of paper indicating the group membership, to be refilled repeatedly until the calculated sample size had been reached. An assistant not involved in the study otherwise blindly drew an envelope from the respective box and handed it to examiner 1.

#### Main procedure and experimental intervention

Each appointment was scheduled for 45 min. After arrival in the lab, examiner 1 welcomed participants and took them into a meeting room, where he explained the procedure in detail but kept participants blind with respect to the study arms and hypotheses of the study. Participants provided written consent. Afterwards, he took the participant to an experimental area comprising a study room and an observation room. The study room was equipped with a sink, a mirror and the following dental hygiene products: a standard manual toothbrush (Elmex InterX Kurzkopf medium^1^), tooth paste (Elmex Kariesschutz^1^), two types of dental floss (waxed and unwaxed dental floss, Elmex^1^), super floss (Meridol^1^), two types of interdental brushes (Elmex^1^: size 2 and 4) and a cup of water for rinsing. The experimenter adjusted the mirror to the participants’ height and then left them alone and moved to the observation room. From there, he could see the participant via cameras and communicate via a microphone. He asked the participant to clean their teeth with the following words: "Please clean your teeth as thoroughly as possible so that they are completely clean." When the participant began to brush their teeth, the experimenter started a stop watch and afterwards opened the envelope with the group assignment. Depending on the group assignment, he either interrupted the brushing process after 1 min (group 1) with the words “Thank you. Please stop brushing your teeth now.” or waited until the participant terminated brushing at their own discretion (group 2). In the latter case, he noted the time the respective participant spent brushing their teeth. After the termination of brushing, the experimenter took the participant to another room where another experimenter, blinded to the condition of the participant (group 1 or 2), welcomed them and asked them to complete the questionnaire on a tablet computer. After completing the questionnaire, the participants received the promised gratification and were said goodbye.

#### Statistical analyses

The hypothesis that the SPOC scores of group 1 (with interruption) are smaller than those of group 2 (without interruption) was tested by t-tests for independent samples. In case of violation of the assumption of equal variances, the respective adaptation of the t-test, the Welch test [38], was applied. The primary outcome was the total SPOC_d_, and the secondary outcomes were the SPOC_n_ and the SPOC_d_-scores for the inner and outer surfaces and the maxilla and mandible, respectively. Group differences are presented together with Cohen’s [37] effect size d.

### Results

#### Descriptive statistics

Sixteen women and eight men (N = 24) aged between 19 and 33 years (M = 24.2 ± 3.7) participated in the study. The average time to complete the SPOC questionnaire was 227 s (± 52, min: 138 max: 365). Ten participants (men: n = 3, women: n = 7) were randomly assigned to group 1 (tooth brushing interrupted after 1 min); fourteen participants (men: n = 5, women: n = 9) were assigned to group 2 (self-terminated tooth brushing). The distribution of sex was not significantly different between the two groups (χ^2^(1) = 0.086, *p* = 0.770). Participants in group 1 brushed their teeth exactly 1 min. None stopped brushing before and all adhered to the examiner’s interruption after 1 min. Participants in group 2 brushed their teeth for at least 104 s (M = 332 ± 127; min = 104, max = 520).

#### Sensitivity of SPOC regarding different oral hygiene behaviour

Since the assumption of variance homogeneity had to be rejected in all cases, Welch tests were computed. Table 3 shows the results of the group comparisons conducted based on participants with a positive comprehension check. Groups differed significantly with respect to the primary outcome SPOC_d_ (*p* = 0.003). All other comparisons revealed large effect sizes, as well (all *p*'s ≤ 0.034).

**Table 3:** Self-perceived oral cleanliness between group 1 (n = 9) and group 2 (n = 10)

Variable	Group 1	Group2	*t *(*df*)	Cohen’s *d*	95% CI for effect size	
	*M* ± *SD*	*M* ± *SD*			Lower	Upper
SPOC_n_	59.6 ± 32.3	83.0 ± 12.4	− 2.046 (10.111)	− 0.980	− 1.925	− 0.009
SPOC_d_						
Total	45.0 ± 32.3	79.7 ± 13.4	− 3.004 (10.451)	− 1.436	− 2.439	− 0.399
Outer surfaces	54.7 ± 38.6	84.0 ± 12.8	− 2.170 (9.591)	− 1.042	− 1.995	− 0.064
Inner surfaces	35.2 ± 33.7	75.5 ± 17.7	− 3.204 (11.801)	− 1.520	− 2.536	− 0.470
Maxilla	39.7 ± 33.9	80.3 ± 14.2	− 3.336 (10.477)	− 1.594	− 2.623	− 0.531
Mandible	50.2 ± 32.8	79.2 ± 13.5	− 2.467 (10.417)	− 1.179	− 2.147	− 0.182

Group 1: Tooth brushing terminated after 1 min. Group 2: Self terminated tooth brushing. SPOC: Self-perceived oral cleanliness. SPOC: Self-perceived oral cleanliness. SPOC_n_: Naïve overall SPOC before being informed within the questionnaire about the standards of a dentist. SPOC_d_: SPOC after being informed about the standards of a dentist. Total: SPOC_d_: Mean of the SPOC_d_-scores for all 12 areas of the dentition (sextants by inner vs. outer surfaces). Outer/inner: SPOC_d_: Mean of the SPOC_d_-scores for outer/inner surfaces. Maxilla/mandible: Mean of the SPOC_d_-scores for the inner and outer surfaces of the maxilla/mandible

In total, 5 of the 24 participants (21%) did not pass the comprehension check (n = 1 in group 1; n = 4 in group 2). Comparing the participants assigned to group 2 with positive and negative comprehension checks revealed no significant differences for SPOC_n_ (*p* = 0.941, d = 0.042) and SPOC_d_ (*p* = 0.822, d = − 0.151). A replication of the analyses including participants with incorrect answers showed comparable results to those reported in Table 3 (− 1.038 ≤ d ≤ − 1.556; all *p*'s ≤ 0.026).

### Discussion

The primary goal of Study 3 was to investigate the sensitivity of the questionnaire to a behavioural manipulation. The results in Table 2 clearly show that participants who had brushed their teeth with a time limit rated the achieved cleanliness significantly lower than those who had brushed without a time limit, regardless of whether cleanliness was considered for the entire mouth or partial areas (outer surfaces/ inner surfaces/ mandible/ maxilla). The instrument is thus sensitive to different behaviour. However, 21% of the participants did not pass the comprehension check. Interestingly, this percentage corresponds to that observed in adolescents and parents (Study 2). This and other issues will now be addressed in the general discussion.

## General discussion

The basis for the motivation for health behaviour change is the awareness that a behavioural change would positively affect one’s health (see Fig. 1). In terms of the quality of oral hygiene, people must be aware of deficits regarding their oral hygiene skills and/or performance in order to consider improving them (see Fig. 2). On the other hand, if they overestimate their performance, this would likely impede their motivation to improve it. This might explain why even people who brush their teeth twice a day and thus appear to be motivated to perform oral hygiene do not engage in oral hygiene skills improvement even if they show insufficient capacity to achieve oral cleanliness [15]. It would thus be helpful to know more about people’s own estimation regarding their oral cleanliness after tooth brushing. It would also be important to understand if their naïve conception of their oral cleanliness would be more realistic if they learn to apply the standards of a dentist. To date, no standardized measurement instrument is available that captures these aspects of self-perceived oral cleanliness (SPOC). The present research thus aimed to develop such an instrument and to examine its psychometric characteristics and validity criteria. The newly developed instrument is called the SPOC questionnaire and assesses two main constructs, the naïve SPOC (SPOC_n_) and that based on the standards of a dentist (SPOC_d_).

Studies 1 and 2 prove that the SPOC questionnaire has good reliability (in terms of internal consistency) and discriminant validity regarding oral hygiene related self-efficacy expectations and the stage of change of daily tooth brushing. Furthermore, SPOC_n_ and SPOC_d_ prove to be different although related constructs. Additionally, Study 3 showed that the SPOC questionnaire is sensitive to differences in oral hygiene behaviour. In summary, the three studies show that the SPOC questionnaire is both a reliable and valid instrument for the assessment of self-perceived oral cleanliness. The results further demonstrate that it is well accepted by participants of different age groups who rate its comprehensibility and feasibility positively.

However, Studies 2 and 3 reveal that not all participants succeeded in learning what standards a dentist would apply to determine oral cleanliness. The significant difference between SPOC_n_ and SPOC_d_ found for participants with a positive comprehension check but not for those with a negative one indicates that this mirrors an actual comprehension problem rather than accidental errors. Interestingly, the percentage of those not passing the comprehension check appears not to depend on age or educational background of the participants: It was more or less the same in a group of adolescents, in parents with diverse educational backgrounds, and in university students. Research from cognitive psychology and science education on conceptual change may provide an explanation for these comprehension difficulties. This research indicates that prior knowledge [39] and naïve ideas [40] can lead to misconceptions that contradict scientific explanations [41, 42] and impede conceptual change [43]. The comprehension check within the SPOC questionnaire could help identify people reluctant to conceptual change with regard to oral cleanliness standards. They could then be targeted to facilitate their conceptual change [43]. Thus, the failure to pass the comprehension check appears to add important information rather than questioning the validity of the instrument. This is especially true since further analysis (partly presented in the Additional file 2) showed that the results of the comprehension check did not affect other important findings of the present research.

One of these findings is that the adolescents and parents investigated in Study 2 considerably overestimated their achieved cleanliness after tooth brushing. Although plaque was not assessed in Studies 1 and 3, based on the results of earlier studies, a similarly pronounced overestimation is likely here as well [15–19]. These results and the limited correlation with actual oral cleanliness support the aforementioned assumption that patients might lack an awareness of their deficits regarding oral hygiene skills. This illustrates the use and need for this newly developed instrument. It offers both the researcher and the clinician the opportunity to assess patients' awareness of their own oral hygiene skills and/or performance. If there is a lack of awareness, a first step to motivate patients to change their behaviour would be to raise this awareness (cf. Fig. 2 and [24]). This can be done by giving the patients feedback regarding the discrepancy between self-perceived oral cleanliness and actual oral cleanliness. The SPOC questionnaire can help to develop a common understanding of the goal of tooth brushing (i.e., plaque removal even at the gingival margin) and to negotiate specific achievable and evaluable goals of oral hygiene training [44, 45].

Interestingly, even though parents also overestimated their oral cleanliness, their estimation correlated moderately with objective plaque levels, while no such associations emerged in adolescents. Additionally, some of their behavioural data correlated with the SPOC scores, while this was not the case in adolescents. This might indicate that adolescents do not have a good awareness of certain aspects of their oral hygiene behaviour (e.g., the degree to which they reach all inner surfaces). In parents, the actual tooth brushing behaviour at inner surfaces was moderately related to all SPOC measures. This, and the fact that behavioural manipulation affected SPOC in Study 3, indicates that at least adults appear to be able to relate their oral cleanliness to their behaviour.

Taken together, the results of the three studies with different sampling approaches indicate good external validity. In terms of the nomological network [46] the different validity measures collected in the three studies also indicate the construct validity of the SPOC questionnaire.

Even though the questionnaire measuring self-perceived oral cleanliness has proven to be reliable and valid, some limitations need to be mentioned. The first limitation relates to Study 1. Since relatives and friends of the staff of the Institute of Medical Psychology had worked on the questionnaire, social desirability could have played a role in answering the questionnaire. However, since the survey was conducted anonymously, the risk of this bias is considered to be rather small. The next limitation refers to Study 2. Since no significant differences were found between the two age groups of adolescents regarding the SPOC-questionnaire, their data were combined for further analyses. With a larger sample, separate analyses would have been preferable. Also the premature termination of participant enrolment in Study 3 is a limitation. However, due to the large effect sizes, statistical significance was achieved even with the substantially reduced sample size. In Study 3 participants of group 1 were interrupted after 1 min of brushing. One might question whether they considered this time as brushing at all. However, all participants answered the SPOC questionnaire that explicitly referred to previous tooth brushing. None of them questioned whether they had brushed teeth at all. Another limitation concerns the participants in the trials themselves. The self-selection of participants may have ensured that they were more likely to be highly motivated, which may have led to an additional overestimation of the ability to achieve oral cleanliness. Furthermore, one might question whether it would be fair to assess patients SPOC with a focus on marginal plaque. More coronal plaque might be easier to estimate for patients. However, it is the marginal plaque that is related to gingivitis and periodontitis [3]. Thus, patients need to develop an awareness about the oral cleanliness achieved in this regard when it comes to the prevention of these diseases.

## Conclusion

The SPOC questionnaire is a reliable and valid instrument to assess patients’ self-perceived oral cleanliness. This instrument allows for the detection of whether patients are aware of deficits regarding their oral hygiene skills and performance, which would be a prerequisite for behavioural change in this regard. It also gives an indication of whether a patient understands the oral hygiene standards applied by the dentist or whether he or she is reluctant to adapt their own concept of oral cleanliness to this standard. This information is useful both at the patient level and for future research. On the patient level, the information provided by the questionnaire might help to target behavioural interventions and to provide feedback. For research, the instrument allows for the assessment of important additional information, helping to better understand the origin of oral hygiene deficits.

## Supplementary Information

**Additional file 1. Title of Data**: The SPOC-Questionnaire. Description of Data: Screenshots of the full questionnaire as it is delivered by the platform SoSci Survey

**Additional file 2. Title of Data**: Supplementary information regarding methods and results of Study 1–3. Description of Data: Study 1 (Assessment of oral hygiene related self-efficacy expectations [OHSEE]; Item analysis of SPOC_d_). Study 2 (Results for the whole group irrespective of the results of the comprehension check; Assessment of stage of change regarding thorough daily oral hygiene [SofC]). Study 3 (Flowchart of the recruitment)

## Data Availability

The datasets used and/or analyzed during the current study are available from the corresponding author on reasonable request. However, for privacy reasons, no individual data allowing identification of participants (e.g., videos) can be provided.

## Abbreviations

ICC: Intraclass correlation
MPI: Marginal Plaque Index
OHSEE: Oral hygiene related self-efficacy expectations
QIT-S: Quality index of tooth brushing regarding brushing time in sextants
RCT: Randomized controlled trial
SofC: Stage of change with respect to thorough daily oral hygiene
SPSS: Statistical package for the social sciences
SPOC: Self-perceived oral cleanliness
SPOC_n_: Naïve overall SPOC before being informed within the questionnaire about the standards of a dentist
SPOC_d_: SPOC after being informed about the standards of a dentist

## Footnotes

1 CP GABA GmbH, Hamburg, Germany.
