# Supplementary material for: Patients’ awareness regarding the quality of their oral hygiene: development and validation of a new measurement instrument
Source: BMC Oral Health. 2022 Dec 22;22:629. doi: 10.1186/s12903-022-02659-4 (PMC9773685; doi:10.1186/s12903-022-02659-4)
Supplement: Supplementary file 1 — Additional file 1. Title of Data: The SPOC-Questionnaire. Description of Data: Screenshots of the full questionnaire as it is delivered by the platform SoSci Survey [file 12903_2022_2659_MOESM1_ESM.pdf]

Additional file 1 for the Article

Patients' awareness regarding the quality of their oral hygiene –development and validation of a new measurement instrument

THE SPOC-QUESTIONNAIRE

Screenshots of the full questionnaire as it is delivered by the platform SoSci Survey [28]. English translations are inserted in red letters on pages 2-9 and 24-27. No translations are provided on the other pages as they repeat the content of page 9 but only refer to other locations. Please note that the sequence of the screenshots represents only one example of the possible sequences randomly generated by the survey program (for details see main text).

The xml-file which is necessary to use the SPOC questionnaire within the platform SoSciSurvey [28] will be made available upon reasonable request and when appropriate credit is given to the authors (e.g. by providing this publication as reference). Upon reasonable request and according to their possibilities the authors will also assist with the use on other platforms and/or tranlsations into other languages.

SPOC-QUESTIONNAIRE (German version)

JUSTUS-LIEBIG-UNIVERSITÄT GIESSEN

4% ausgefüllt

Im Folgenden finden Sie verschiedene Fragen zum Zähneputzen. In the following, you will find several questions about tooth brushing.

Beachten Sie bitte: Es gibt **keine richtigen oder falschen Antworten** und **keine guten oder schlechten**. Please note: there are no right or wrong answers and no good or bad ones.

Wichtig ist, dass Sie auswählen, was für Sie am besten zutrifft. It is important that you choose what applies best to you.

Weiter next

Justus-Liebig-Universität Gießen – 2019

JUSTUS-LIEBIG-UNIVERSITÄT GIESSEN

4% ausgefüllt

Schätzen Sie bitte ein, wie sauber Sie gerade Ihre Zähne geputzt haben. **Please estimate how clean you have just brushed your teeth.**

Bewegen Sie den Schieberegler, um Ihre Einschätzung zwischen „gar nicht sauber“ und „ganz sauber“ abzugeben. Move the slider to give your estimation between “not clean at all” and “completely clean”.

Meine Zähne sind jetzt... **My teeth are now...**

gar nicht sauber ———— ganz sauber

not clean at all — completely clean

Weiter **next**

JUSTUS-LIEBIG-UNIVERSITÄT GIESSEN

7% ausgefüllt

Ziel des Zähneputzens ist es, die Zähne vollständig von Zahnbelägen (Plaque) zu befreien. The goal of tooth brushing is the complete removal of dental deposits (plaque) from the teeth.

Auch wenn sich Zahnbeläge nur am Zahnfleischrand befinden, ist ein Zahn nicht sauber. Even if deposits only appear at the gum line, the tooth is not clean.

Alle drei abgebildeten Zähne sind nicht sauber. None of the three depicted teeth are clean.

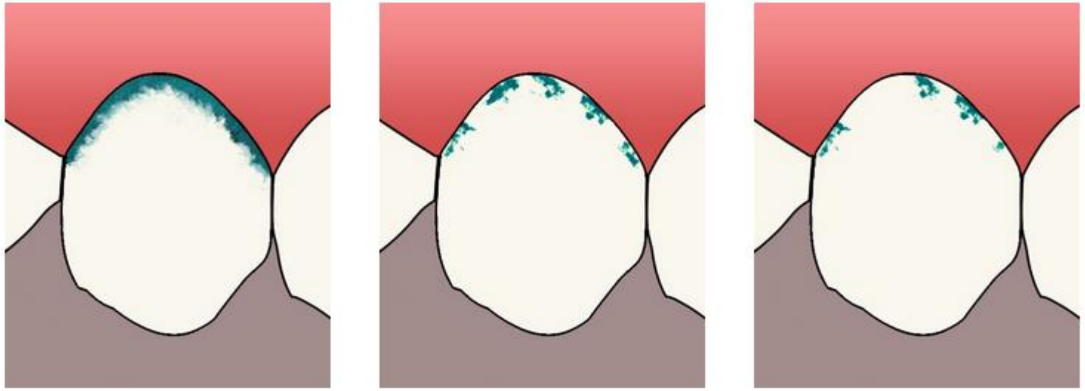

Weiter **next**

JUSTUS-LIEBIG-UNIVERSITÄT GIESSEN

11% ausgefüllt

Auf der folgenden Abbildung sehen Sie nun, wie solche Zahnbeläge erfasst werden:
The following illustration shows how such dental deposits are recorded:

- Der Bereich am Zahnfleischrand wird in vier Felder unterteilt.
  The area at the gum line is divided into four sections.
- Für jedes **einzelne** Feld wird notiert, ob sich noch Belag darin befindet oder nicht.
  For each **single** section, it is noted whether it still contains dental deposits or not.
- Dies erfolgt sowohl an den Zahnaußenflächen als auch an den Zahninnenflächen.
  This is done for both the outer surfaces of the teeth and the inner surfaces of the teeth.

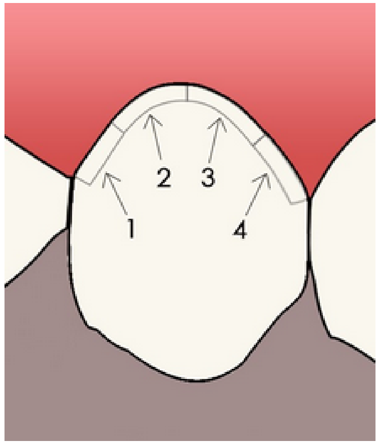

Weiter **next**

JUSTUS-LIEBIG-
UNIVERSITÄT
GIESSEN

15% ausgefüllt

Das Ergebnis der Untersuchung könnte z. B. so aussehen: For example, the result of the examination might be:

KEIN Feld sauber
NO section clean

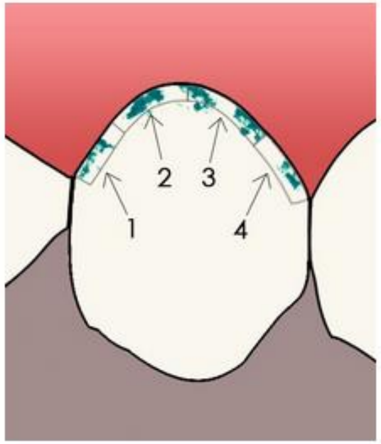

Feld Nummer 2 sauber
Section number 2 clean

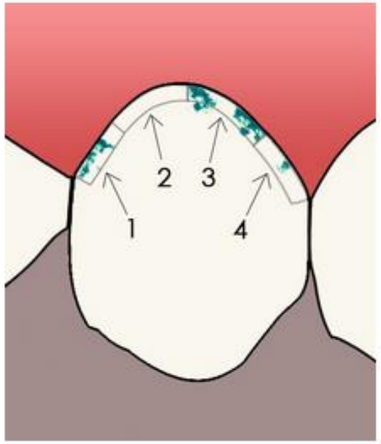

ALLE Felder sauber
ALL sections clean

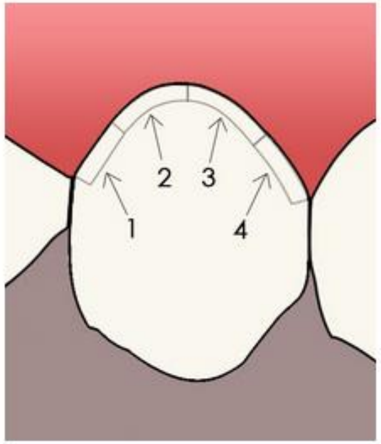

Weiter **next**

JUSTUS-LIEBIG-UNIVERSITÄT GIESSEN

19% ausgefüllt

Was denken Sie: Wie sauber haben Sie gerade ihre Zähne geputzt? Haben Sie alle Felder erreicht?
What do you think: How clean did you just brush your teeth? Did you reach all the sections?
Im Folgenden werden nacheinander verschiedene Bereiche in Ihrem Mund abgefragt:
In the following, different areas in your mouth are queried one after the other:

- Ober- und Unterkiefer upper and lower jaw
- Außen- und Innenflächen der Zähne outer and inner surfaces of the teeth
- rechte und linke Seite right and left side
- Backenzähne und vordere Zähne back teeth and front teeth

Bitte achten Sie darauf, immer nur zu diesem Bereich zu antworten! Please make sure to answer only on this area at a time!

Weiter **next**

JUSTUS-LIEBIG-UNIVERSITÄT GIESSEN

22% ausgefüllt

Jetzt geht es um die **Innenseiten** Ihrer Zähne im **Unterkiefer**.

Now it is about the **inner surfaces** of your teeth in the **lower jaw**.

Weiter **next**

JUSTUS-LIEBIG-UNIVERSITÄT GIESSEN

26% ausgefüllt

Innenflächen Unterkiefer
inner surfaces lower jaw

Backenzähne rechts
back teeth right

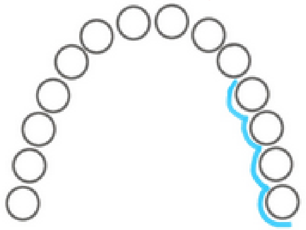

Bei **4** Feldern pro Zahn ergeben sich für **4** Backenzähne **16** Felder.
With **4** sections per tooth, this results in **16** sections for **4** teeth.

Estimate how many sections on the inside of the lower right teeth you just got clean.

Schätzen Sie ein, wieviele Felder Sie gerade unten rechts von innen sauber geputzt haben.

Bewegen Sie den Schieberegler, um Ihre Einschätzung zwischen „keines sauber" und „alle sauber" abzugeben. Move the slider to give your estimation between "none clean" and "all clean".

Saubere Felder Clean sections

keines sauber — alle sauber

none clean — all clean

Weiter next

JUSTUS-LIEBIG-UNIVERSITÄT GIESSEN

30% ausgefüllt

Innenflächen Unterkiefer

vordere Zähne

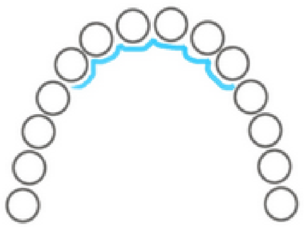

Bei **4** Feldern pro Zahn ergeben sich für **6** vordere Zähne **24** Felder.

Schätzen Sie ein, wieviele Felder Sie gerade bei den unteren Vorderzähnen von innen sauber geputzt haben.

Bewegen Sie den Schieberegler, um Ihre Einschätzung zwischen „keines sauber“ und „alle sauber“ abzugeben.

Saubere Felder

keines sauber ——— alle sauber

Weiter

JUSTUS-LIEBIG-UNIVERSITÄT GIESSEN

33% ausgefüllt

Innenflächen Unterkiefer

Backenzähne links

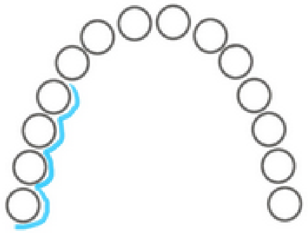

Bei **4** Feldern pro Zahn ergeben sich für **4** Backenzähne **16** Felder.

Schätzen Sie ein, wieviele Felder Sie gerade unten links von innen sauber geputzt haben.
Bewegen Sie den Schieberegler, um Ihre Einschätzung zwischen „keines sauber“ und „alle sauber“ abzugeben.

Saubere Felder

keines sauber ——— alle sauber

Weiter

JUSTUS-LIEBIG-UNIVERSITÄT GIESSEN

37% ausgefüllt

Jetzt geht es um die **Außenseiten** Ihrer Zähne im **Unterkiefer.**

Weiter

JUSTUS-LIEBIG-UNIVERSITÄT GIESSEN

41% ausgefüllt

Außenflächen Unterkiefer

Backenzähne rechts

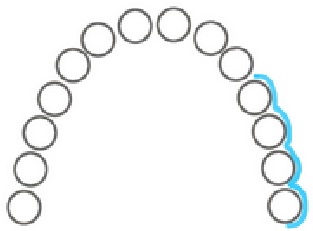

Bei **4** Feldern pro Zahn ergeben sich für **4** Backenzähne **16** Felder.

Schätzen Sie ein, wieviele Felder Sie gerade unten rechts von außen sauber geputzt haben.
Bewegen Sie den Schieberegler, um Ihre Einschätzung zwischen „keines sauber“ und „alle sauber“ abzugeben.

Saubere Felder

keines sauber — alle sauber

Weiter

JUSTUS-LIEBIG-UNIVERSITÄT GIESSEN

44% ausgefüllt

Außenflächen Unterkiefer

vordere Zähne

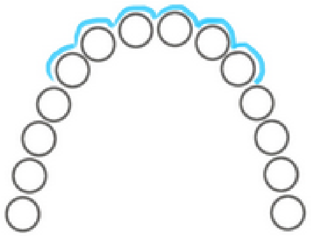

Bei **4** Feldern pro Zahn ergeben sich für **6** vordere Zähne **24** Felder.

Schätzen Sie ein, wieviele Felder Sie gerade bei den unteren Vorderzähnen von außen sauber geputzt haben.

Bewegen Sie den Schieberegler, um Ihre Einschätzung zwischen „keines sauber“ und „alle sauber“ abzugeben.

Saubere Felder

keines sauber —— alle sauber

Weiter

JUSTUS-LIEBIG-UNIVERSITÄT GIESSEN

48% ausgefüllt

Außenflächen Unterkiefer

Backenzähne links

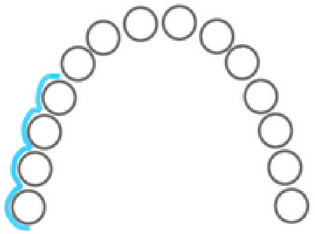

Bei **4** Feldern pro Zahn ergeben sich für **4** Backenzähne **16** Felder.

Schätzen Sie ein, wieviele Felder Sie gerade unten links von außen sauber geputzt haben.

Bewegen Sie den Schieberegler, um Ihre Einschätzung zwischen „keines sauber“ und „alle sauber“ abzugeben.

Saubere Felder

keines sauber — alle sauber

Weiter

Justus Liebig Universität Gießen – 2019

JUSTUS-LIEBIG-UNIVERSITÄT GIESSEN

52% ausgefüllt

Jetzt geht es um die **Innenseiten** Ihrer Zähne im **Oberkiefer**.

Weiter

JUSTUS-LIEBIG-UNIVERSITÄT GIESSEN

56% ausgefüllt

Innenflächen Oberkiefer

Backenzähne rechts

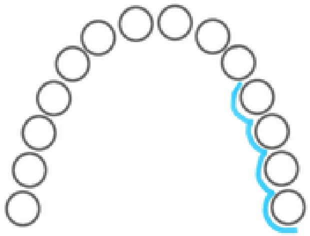

Bei **4** Feldern pro Zahn ergeben sich für **4** Backenzähne **16** Felder.

Schätzen Sie ein, wieviele Felder Sie gerade oben rechts von innen sauber geputzt haben.
Bewegen Sie den Schieberegler, um Ihre Einschätzung zwischen „keines sauber“ und „alle sauber“ abzugeben.

Saubere Felder

keines sauber ——— alle sauber

Weiter

JUSTUS-LIEBIG-UNIVERSITÄT GIESSEN

59% ausgefüllt

Innenflächen Oberkiefer

vordere Zähne

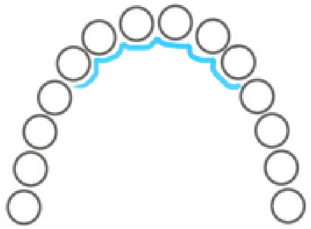

Bei **4** Feldern pro Zahn ergeben sich für **6** vordere Zähne **24** Felder.

Schätzen Sie ein, wieviele Felder Sie gerade bei den oberen Vorderzähnen von innen sauber geputzt haben.

Bewegen Sie den Schieberegler, um Ihre Einschätzung zwischen „keines sauber“ und „alle sauber“ abzugeben.

Saubere Felder

keines sauber ——— alle sauber

Weiter

JUSTUS-LIEBIG-UNIVERSITÄT GIESSEN

63% ausgefüllt

Innenflächen Oberkiefer

Backenzähne links

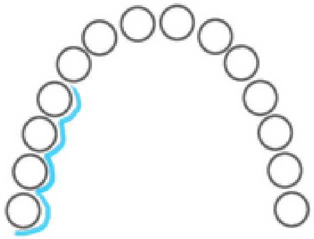

Bei **4** Feldern pro Zahn ergeben sich für **4** Backenzähne **16** Felder.

Schätzen Sie ein, wieviele Felder Sie gerade oben links von innen sauber geputzt haben.
Bewegen Sie den Schieberegler, um Ihre Einschätzung zwischen „keines sauber“ und „alle sauber“ abzugeben.

Saubere Felder

keines sauber ———— alle sauber

Weiter

Justus Liebig Universität Gießen – 2019

JUSTUS-LIEBIG-
UNIVERSITÄT
GIESSEN

67% ausgefüllt

Jetzt geht es um die **Außenseiten** Ihrer Zähne im **Oberkiefer**.

Weiter

JUSTUS-LIEBIG-UNIVERSITÄT GIESSEN

70% ausgefüllt

Außenflächen Oberkiefer

Backenzähne rechts

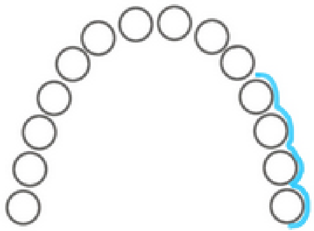

Bei **4** Feldern pro Zahn ergeben sich für **4** Backenzähne **16** Felder.

Schätzen Sie ein, wieviele Felder Sie gerade oben rechts von außen sauber geputzt haben.
Bewegen Sie den Schieberegler, um Ihre Einschätzung zwischen „keines sauber" und „alle sauber" abzugeben.

Saubere Felder

keines sauber — alle sauber

Weiter

JUSTUS-LIEBIG-UNIVERSITÄT GIESSEN

74% ausgefüllt

Außenflächen Oberkiefer

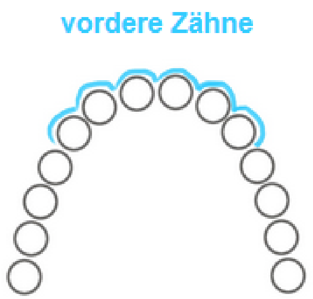

Bei **4** Feldern pro Zahn ergeben sich für **6** vordere Zähne **24** Felder.

Schätzen Sie ein, wieviele Felder Sie gerade bei den oberen Vorderzähnen von außen sauber geputzt haben.

Bewegen Sie den Schieberegler, um Ihre Einschätzung zwischen „keines sauber“ und „alle sauber“ abzugeben.

Saubere Felder

keines sauber — alle sauber

Weiter

JUSTUS-LIEBIG-UNIVERSITÄT GIESSEN

78% ausgefüllt

Außenflächen Oberkiefer

Backenzähne links

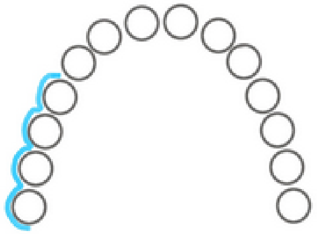

Bei **4** Feldern pro Zahn ergeben sich für **4** Backenzähne **16** Felder.

Schätzen Sie ein, wieviele Felder Sie gerade oben links von außen sauber geputzt haben.

Bewegen Sie den Schieberegler, um Ihre Einschätzung zwischen „keines sauber“ und „alle sauber“ abzugeben.

Saubere Felder

keines sauber — alle sauber

Weiter

JUSTUS-LIEBIG-
UNIVERSITÄT
GIESSEN

81% ausgefüllt

Nachfolgend sehen Sie ein paar Bilder von unterschiedlich sauber geputzen Zähnen.

In the following you will find a few pictures of differently clean teeth.

Bitte geben Sie an, wie viele **saubere** Felder der Zahn hat.

Please indicate how many **clean** sections the tooth has.

Weiter **next**

JUSTUS-LIEBIG-UNIVERSITÄT GIESSEN

85% ausgefüllt

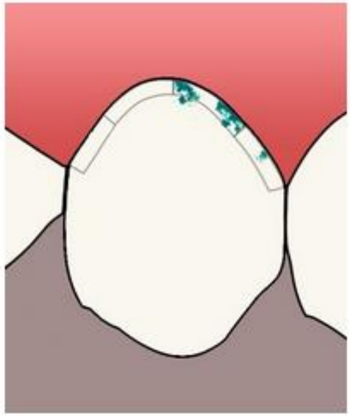

Wieviel saubere Felder hat der abgebildete Zahn? **How many clean sections does the tooth shown have?**

Bitte geben Sie eine Ziffer ein. *Please enter a number.*

Weiter **next**

JUSTUS-LIEBIG-
UNIVERSITÄT
GIESSEN

89% ausgefüllt

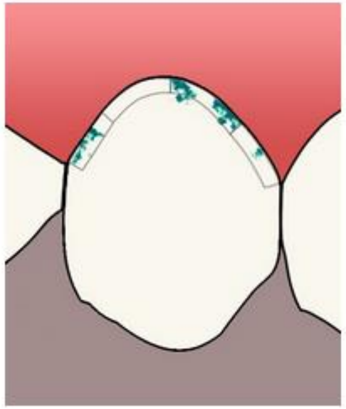

Wieviel saubere Felder hat der abgebildete Zahn? **How many clean sections does the tooth shown have?**

Bitte geben Sie eine Ziffer ein. *Please enter a number.*

Weiter **next**

JUSTUS-LIEBIG-UNIVERSITÄT GIESSEN

93% ausgefüllt

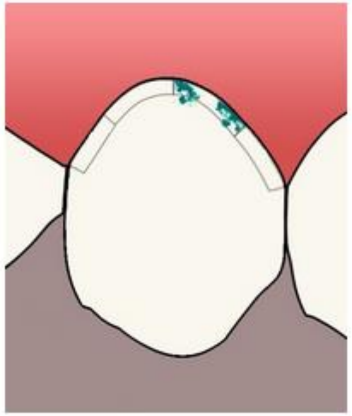

Wieviel saubere Felder hat der abgebildete Zahn? How many clean sections does the tooth shown have?

Bitte geben Sie eine Ziffer ein. *Please enter a number.*

Weiter next
