# Supplementary material for: Patients’ awareness regarding the quality of their oral hygiene: development and validation of a new measurement instrument
Source: BMC Oral Health. 2022 Dec 22;22:629. doi: 10.1186/s12903-022-02659-4 (PMC9773685; doi:10.1186/s12903-022-02659-4)
Supplement: Supplementary file 2 — Additional file 2. Title of Data: Supplementary information regarding methods and results of Study 1–3. Description of Data: Study 1 (Assessment of oral hygiene related self-efficacy expectations [OHSEE]; Item analysis of SPOCd). Study 2 (Results for the whole group irrespective of the results of the comprehension check; Assessment of stage of change regarding thorough daily oral hygiene [SofC]). Study 3 (Flowchart of the recruitment) [file 12903_2022_2659_MOESM2_ESM.pdf]

Additional file 2 for the Article

Patients' awareness regarding the quality of their oral hygiene – development and validation of a new measurement instrument

SUPPLEMENTARY INFORMATION REGARDING METHODS AND RESULTS OF STUDY 1-3

Study 1: Page 1

Study 2: Page 2-5

Study 3: Page 6

Study 1

Assessment of oral hygiene related self-efficacy expectations (OHSEE)

Table S1. Adaptation of the scale of Stewart et al. [31] to assess oral hygiene related self-efficacy expectations (OHSEE)[#]

German version	English translation
Ich sehe mich in der Lage, meine Zähne einmal täglich gründlich zu reinigen, …	I find myself able to clean my teeth thoroughly once a day ...
wenn alles normal läuft.	when things are normal.
wenn ich traurig bin.	when I'm sad.
wenn ich zu tun habe.	when I'm busy.
wenn ich im Stress bin.	when I'm stressed.
wenn ich müde bin.	when I'm tired.
wenn ich im Urlaub bin.	when I'm on vacation.
wenn ich dafür etwas Schönes unterbrechen muss.	when I have to stop something enjoyable.
nachdem ich längere Zeit nachlässig war.	after having been careless for a long time.
wenn ich keine Lust dazu habe.	when I don't feel like it.

[#]Answer format: 5-point Likert scale from *1 = trifft überhaupt nicht zu [not at all true] to 5 = trifft voll und ganz zu [totally true]*

Item analysis of $SPOC_d$

Table S2. Item analysis of self-perceived oral cleanliness after beeing informed about the standards of a dentist ($SPOC_d$) (N=56)

Item[#]	Min	Max	*M*	*SD*	Skew	Kurtosis	Item-Total Correlation	Cronbach's α if item deleted
Estimate how many sections you just have cleaned on the…								
outside of the upper right teeth.	38	100	79.8	13.7	–0.571	0.112	.69	.934
outside of the upper front teeth.	54	100	87.4	10.9	–1.031	0.550	.51	.939
outside of the upper left teeth.	22	100	78.6	15.3	–1.435	3.285	.82	.929
outside of the lower right teeth.	28	100	80.1	14.9	–1.236	2.279	.74	.932
outside of the lower front teeth.	19	100	85.6	14.7	–2.194	6.814	.59	.937
outside of the lower left teeth.	24	100	80.3	15.5	–1.190	2.722	.68	.934
inside of the upper right teeth.	22	100	70.7	16.1	–0.604	0.644	.73	.932
inside of the upper front teeth.	2	100	70.1	23.0	–0.943	0.864	.83	.929
inside of the upper left teeth.	2	100	70.7	18.3	–0.898	2.031	.80	.930
inside of the lower right teeth.	8	100	73.0	18.6	–1.223	2.292	.87	.926
inside of the lower front teeth.	15	100	70.3	23.3	–0.577	–0.450	.74	.934
inside of the lower left teeth.	24	100	74.4	17.2	–1.007	1.384	.75	.931

[#]translated from German; for German version see Online resource 1. Answer format: visual analogue scale ranging from none (0) to all (100)

Study 2

Results for the whole group irrespective of the results of the comprehension check

The following analyses refer to all participants, i.e. without excluding those participants with a negative comprehension check.

SPOC-descriptives and internal consistency

Table S3 shows the descriptives of $SPOC_n$ and various aggregated $SPOC_d$ scores for the three age groups. Again, the two adolescent age groups do not differ in $SPOC_n$- and $SPOC_d$-scores (all p's ≥ .312) so they are analysed as one group again. The item-total correlations of the $SPOC_d$-items varied between r =.63 and r = .89 in adolescents and r = .72 and r = .88 in adults. Cronbach's α for $SPOC_d$ equaled α = .958 in adolescents and α = .955 in parents, respectively. Table 4 shows Cronbach's α if item deleted.

Table S3. Descriptives of $SPOC_n$ and various aggregated $SPOC_d$ scores of parents and adolescents

Item	10-year-olds (n = 42)<br>*M ± SD*	15-year-olds (n = 24)<br>*M ± SD*	parents (n = 66)<br>*M ± SD*
$SPOC_n$	76.9 ± 17.2	81.0 ± 13.2	75.5 ± 17.9
$SPOC_d$			
total	70.6 ± 19.7	68.7 ± 14.5	70.8 ± 16.9
outer	76.0 ± 18.2	75.3 ± 14.4	76.1 ± 16.9
inner	65.2 ± 23.5	62.0 ± 18.1	65.5 ± 18.6
maxilla	71.8 ± 18.9	70.9 ± 13.9	71.2 ± 17.4
mandible	69.4 ± 20.9	66.5 ± 16.2	70.5 ± 17.4

SPOC: Self-perceived oral cleanliness. $SPOC_n$: Naïve overall SPOC before being informed within the questionnaire about the standards of a dentist. $SPOC_d$: SPOC after being informed about the standards of a dentist. Total: $SPOC_d$: Mean of the $SPOC_d$-scores for all 12 areas of the dentition (sextants by inner vs. outer surfaces). Outer/inner: $SPOC_d$: Mean of the $SPOC_d$-scores for outer/inner surfaces. Maxilla/mandible: Mean of the $SPOC_d$-Scores for the inner and outer surfaces of the maxilla/mandible.

Table S4. Cronbach's α if item is deleted in the sample of parents and adolescents

	Cronbach's α if item deleted	
Item#	adolescents<br>all (n= 66) / 10-year-olds (n=42) / 15-year-olds (n=24)	parents<br>n = 66
Estimate how many sections you just have cleaned on the…		
outside of the upper right teeth.	.956 / .967 / .920	.950
outside of the upper front teeth.	.959 / .966 / .941	.952
outside of the upper left teeth.	.955 / .965 / .922	.951
outside of the lower right teeth.	.954 / .965 / .918	.948
outside of the lower front teeth.	.958 / .968 / .926	.951
outside of the lower left teeth.	.953 / .964 / .917	.950
inside of the upper right teeth.	.953 / .965 / .916	.951
inside of the upper front teeth.	.955 / .965 / .923	.952
inside of the upper left teeth.	.954 / .965 / .920	.950
inside of the lower right teeth.	.952 / .963 / .917	.953
inside of the lower front teeth.	.954 / .964 / .923	.952
inside of the lower left teeth.	.953 / .964 / .919	.950

#translated from German; for German version see Online resource 1. Answer format: visual analogue scale ranging from none (0) to all (100)

Relationship between SPOC and actual oral cleanliness

Within the total group the *de facto* oral cleanliness (100 – MPI) was M = 21.4 ± 15.7 for the adolescents and M = 30.3 ± 15.3 of the parents and differed highly significant from the respective $SPOC_d$ both in adolescents (paired t-test: $p < .001$, $d = 1.947$) and parents ($p < .001$, $d = 2.211$) in the total sample (N=66), as well. As is the analyses limited to those who passed the comprehension tests no meaningful correlations were found between the *de facto* oral cleanliness and the two SPOC scores in adolescents ($SPOC_n$: rho = .047, p = .708; $SPOC_d$: rho = -.033, p = .790) but in parents ($SPOC_n$: rho = .500, p < .001; $SPOC_d$: rho = .375, p = .002).

Relationship between SPOC and tooth brushing behaviour

Regarding relationships to behaviour, analyses of the whole group also resemble results confined to those, who passed the comprehension test. Within the whole group of adolescents (N=66) there were no significant correlations between the two SPOC scores ($SPOC_n$ and $SPOC_d$) and total time of tooth brushing (rho ≤ .150; p ≥ .230). Additionally, no meaningful correlations were observable between the QIT-S scores for inner and outer surfaces and the respective $SPOC_d$ scores (rho ≤ .089; p ≥ .475) or the general $SPOC_d$ (rho ≤.117; p ≥.350) or $SPOC_n$ score (rho ≤ -.073; p ≥ .560).

Within the whole group of parents, a significant correlation was found between the $SPOC_d$ score for inner surfaces and the QIT-S at inner surfaces (rho =.526; p<.001). This QIT-S score also correlated with $SPOC_n$ (rho=.588; p <.001) and the general $SPOC_d$ (rho =.490; p<.001). No significant correlations were seen between the QIT-S at outer surfaces and the respective SPOC score (rho= –.210, p=.091) or the two overall SPOC scores ($SPOC_n$: rho=–.018, p=.887; $SPOC_d$: rho = –.111; p=.373). Total tooth brushing time did not significantly correlate with $SPOC_n$ (rho=.164, p=.189) or $SPOC_d$ (rho = -.028; p=.822).

Assessment of stage of change regarding thorough daily oral hygiene (SofC)

Table S5. Items of the Stage of Change (Prochaska & DiClemente [33]) assessment

German version	English translation
Ich sorge täglich für saubere Zähne.	I keep my teeth clean every day.
[1] Nein, und ich habe es auch nicht innerhalb der nächsten sechs Monate vor.	[1] No, and I don't plan to do so within the next six months.
[2] Nein, aber ich habe es innerhalb der nächsten sechs Monate vor.	[2] No, but I plan to do it within the next six months.
[3] Nein, aber ich habe es innerhalb der nächsten 30 Tage vor.	[3] No, but I plan to do so within the next 30 days.
[4] Ja, aber seit weniger als sechs Monaten.	[4] Yes, but for less than six months.
[5] Ja, und das schon seit mehr als sechs Monaten.	[5] Yes, for more than six months now.

Stage of Change: [1] Absichtslosigkeit (Precontemplation), [2] Absichtsbildung (Contemplation), [3] Vorbereitung (Preparation), [4] Handlung (Action), [5] Aufrechterhaltung (Maintenance)

Study 3

Flowchart of the recruitment

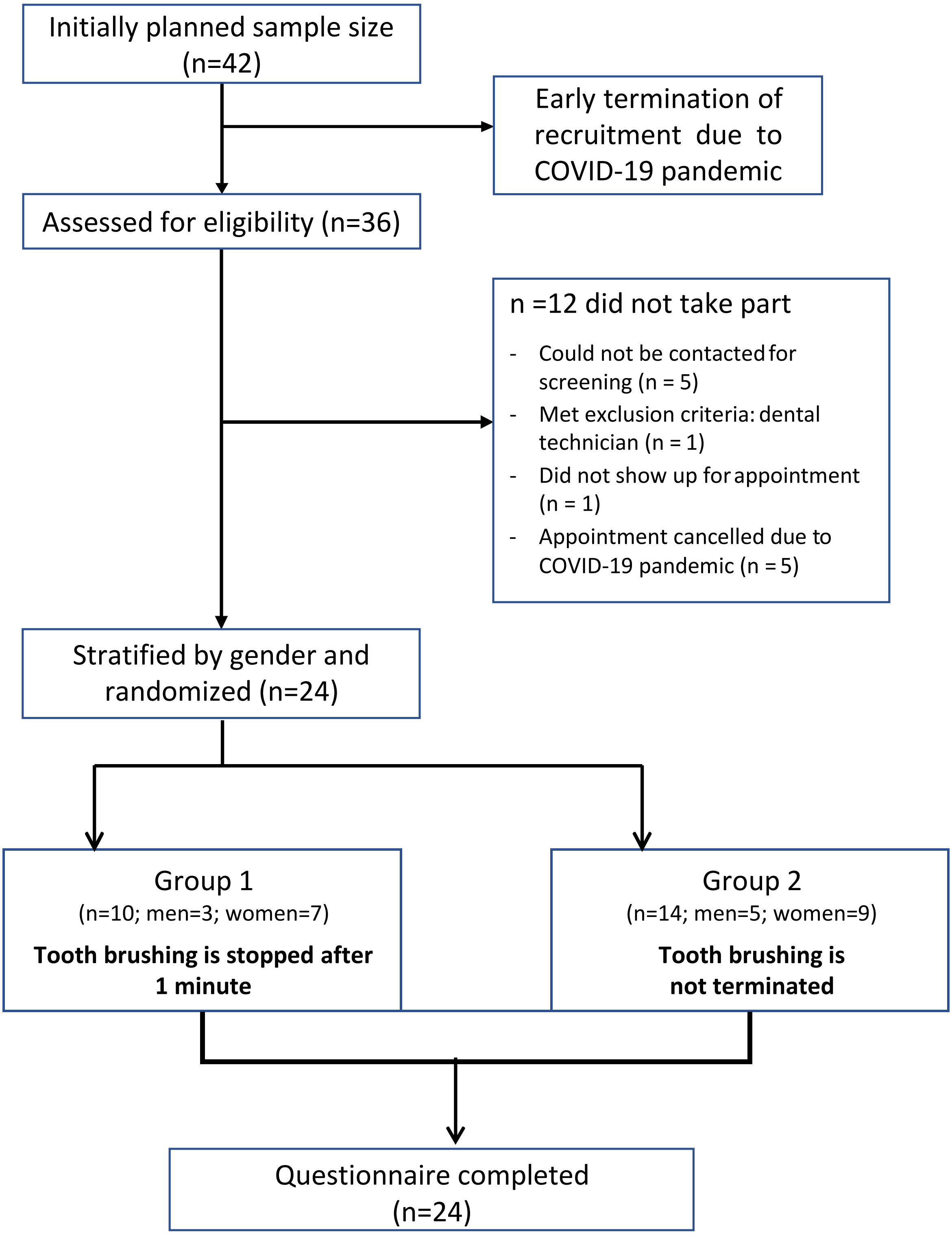

Figure S1. Flowchart of the recruitment in Study 3
